# Analytical Equations for the Prediction of the Failure Mode of Reinforced Concrete Beam–Column Joints Based on Interpretable Machine Learning and SHAP Values

**DOI:** 10.3390/s24247955

**Published:** 2024-12-12

**Authors:** Ioannis Karampinis, Martha Karabini, Theodoros Rousakis, Lazaros Iliadis, Athanasios Karabinis

**Affiliations:** 1 Laboratory of Mathematics and Informatics (ISCE), Department of Civil Engineering, Democritus University of Thrace, 67100 Xanthi, Greece; ikarampi@civil.duth.gr (I.K.); liliadis@civil.duth.gr (L.I.); 2 Laboratory of Reinforced Concrete and Seismic Design, Department of Civil Engineering, Democritus University of Thrace, 67100 Xanthi, Greece; mkarampi@civil.duth.gr (M.K.); trousak@civil.duth.gr (T.R.)

**Keywords:** reinforced concrete, beam–column joints, failure mode prediction, machine learning, SHAP, analytical equations

## Abstract

One of the most critical components of reinforced concrete structures are beam–column joint systems, which greatly affect the overall behavior of a structure during a major seismic event. According to modern design codes, if the system fails, it should fail due to the flexural yielding of the beam and not due to the shear failure of the joint. Thus, a reliable tool is required for the prediction of the failure mode of the joints in a preexisting population of structures. In the present paper, a novel methodology for the derivation of analytical equations for this task is presented. The formulation is based on SHapley Additive exPlanations values, which are commonly employed as an explainability tool in machine learning. Instead, in the present paper, they were also utilized as a transformed target variable to which the analytical curves were fitted, which approximated the predictions of an underlying machine learning model. A dataset comprising 478 experimental results was utilized and the eXtreme Gradient Boosting algorithm was initially fitted. This achieved an overall accuracy of ≈84%. The derived analytical equations achieved an accuracy of ≈78%. The corresponding metrics of precision, recall, and the F1-score ranged from ≈76% to ≈80% and were close across the two modes, indicating an unbiased model.

## 1. Introduction

One of the most critical aspects that affect the overall behavior of a reinforced concrete (RC) structure during a major seismic event pertains to the mechanical behavior of its beam–column joints [1]. Joints support adjacent beams and slabs and, thus, their failure can lead to structural destabilization and part of the structure collapsing, potentially endangering human lives [2]. This mechanical behavior is largely described by two distinct and complementary aspects. On the one hand, the total strength of the joint provides a quantification of the capacity of the system in terms of shear forces. On the other hand, the qualitative behavior of the joint in terms of its failure mode can be an even more important factor in terms of the overall safety of the system and the amount of seismic energy it can absorb.

Qualitatively, there are two primary failure modes that an RC beam–column joint system can exhibit [3]. In the first one, the longitudinal reinforcement bars in the adjacent beam are the first to reach their yield capacity. However, the system maintains its stability and, furthermore, due to a phenomenon known as hardening [4], the beam can absorb further amounts of seismic energy until either the reinforcement bars or the concrete fail [2]. In the second case, the joint fails due to shear prior to the beam reaching the aforementioned yielding point. This failure mode is fundamentally different from the previous one. Contrary to the ductile, yielding failure mode caused by bending, the joint failure due to shear is sudden, brittle, and leads to the destabilization of the system. In addition, no further amounts of energy can be absorbed.

To this end, modern design codes [5,6,7] are often grounded in the so-called principle of ductility, mandating that, if failure is to occur, the first failure mode is to be preferred. Thus, given the large number of existing RC structures, the prediction of the failure modes of their joints is crucial. If a structure does not abide by the standards imposed by modern design codes, preventative steps might be required [8,9]. Thus, significant research efforts have been dedicated to this task, both from a theoretical and an experimental perspective [10,11,12,13].

From a different perspective, the proliferation of machine learning (ML) methodologies has allowed for the implementation of powerful ML algorithms in many engineering challenges, producing state-of-the-art results [14,15]. Specifically for the task of RC beam–column joint failure mode prediction, several researchers have previously examined the problem from different perspectives.

In particular, Kotsovou et al. [16] utilized artificial neural networks (ANNs) to estimate the total strength of exterior joints and predict their failure mode. Similarly, Suwal and Guner also employed ANN algorithms in their work [17]. However, contrary to [16], they worked on a more extensive dataset, which included interior joints as well. Mangathalu and Jeon [18] experimented with various ML algorithms to predict both the strength of a joint as well as its failure mode. This included the implementation of so-called intrinsically explainable models [19], such as LASSO logistic regression, which also produced analytical expressions. However, the results presented therein were unbalanced, as the model was greatly skewed in favor of one of the failure modes. Marie et al. [20] employed several algorithms, including support vector machines (SVMs), ordinary least squares (OLS), and non-parametric kernel regression, to estimate joints’ ultimate bearing capacity. Lastly, Gao and Lin [21] utilized models such as k-nearest neighbors (k-NN), eXtreme Gradient Boosting (XGBoost), ANNs, and random forest to predict an RC beam–column joint failure mode. They also utilized the SHapley Additive exPlanations (SHAP values) explainability algorithm to quantify the overall significance of each of their considered input features in the model predictions.

The research presented herein exhibits notable novel elements. A novel methodology for the derivation of analytical equations for the prediction of the RC joint failure mode is presented. The methodology is based on a recently developed modeling approach [22], which utilizes SHAP values as a basis to form simplified analytical expressions that approximate the predictions of the underlying ML model. SHAP is a feature attribution algorithm. This means that the predictions of the underlying model are decomposed into terms corresponding to the contribution of each individual feature. Thus, it is often employed to quantify the magnitude of the total effect of each feature in an outcome [21]. In the work presented herein, SHAP values were not only employed to gauge the importance of each feature, i.e., to identify which features should be utilized in a potential analytical expression. Rather, SHAP values themselves formed the basis of these analytical expressions.

Therefore, the methodology presented herein offers several distinct advantages in comparison to the currently established literature, such as the LASSO logistic regression mentioned above. Firstly, feature selection was carried out in an informed manner, utilizing SHAP, and components were added to the resulting equations incrementally. Secondly, this allowed for the tradeoff between model complexity and accuracy to be tuned, depending on the specific application at hand. Thirdly, the form of the terms corresponding to each individual feature were adjusted independently. In addition, this was not carried out blindly, but rather in an informed manner, guided by the form of the respective SHAP scatter plots, as it is shown in Section 3.2. Finally, so-called precision–recall (P-R) curves were employed to demonstrate how the resulting equations could be further fine-tuned, depending on the requirements of the application at hand.

## 2. Materials and Methods

### 2.1. Dataset Overview and Feature Engineering

The dataset that was employed in the present study comprised experimental measurements of reinforced concrete beam–column joints. A total of 486 specimens were contained in the dataset, which were originally collected by Suwal and Guner [17] from 153 studies previously published in the literature. The experiments pertained to the cyclic loading of beam–column joint systems until failure occurred, mimicking their behavior during a significant seismic event. In all cases, the applied load at the moment of failure was measured using appropriately placed sensors. In addition, the failure mode of each specimen was also recorded. Thus, each input vector in the original dataset was described by the following 14 attributes [17]:

1. The joint type: This categorical variable described whether the joint was “interior”, i.e., beams are attached on two opposing sides, or “exterior”.
2. $f_{c}$: The compressive strength of the concrete, measured in MPa.
3. $\rho_{jt}$: The amount of transverse reinforcement, i.e., stirrups, in the joint, expressed as a percentage of its area.
4. $f_{yjt}$: The yield strength of the stirrups in the joint, measured in MPa.
5. $\rho_{b},\;\rho_{c}$: The amount of longitudinal reinforcement in the beam and column, respectively, expressed as a percentage of the respective cross-sectional areas.
6. $f_{yb},\;f_{yc}$: The yield strength of the longitudinal reinforcement in the beam and column, respectively, measured in MPa.
7. $h_{b},\;h_{c},\;b_{b},\;b_{c}$: The height (*h*) and width (*b*) of the beam and column, respectively, measured in mm.
8. ALF: The axial load factor, i.e., the axial load applied to the column normalized to the respective compressive strength $f_{c}$.
9. The failure mode: This was the dependent variable in the present study and took two distinct values. Specifically, the value “Joint Shear” (“JS”) corresponded to cases where the joint failed suddenly in a brittle, shear manner and the beam had not yet reached its yield capacity. Accordingly, the value “Beam Yield-Joint Shear” (“BY-JS”) pertained to specimens wherein the ductile flexural yielding of the beam reinforcement preceded the brittle shear failure of the joint [3].

As was stated in the Introduction, the aim of the present study was to obtain simple, analytical expressions to predict the failure mode of the joints based on the independent variables/features. Thus, the input features were critical to the proposed formulation and, to this end, a procedure known as feature engineering [23] was performed on the original dataset. It should be highlighted that feature engineering is more general and more involved than preprocessing a dataset. Indeed, preprocessing steps often include the appropriate scaling of the features [24], removing outliers, or imputing missing values [25]. On the other hand, feature engineering can also include the generation of new features from existing ones, which can be more appropriate for specific modeling purposes.

To this end, in the present study, each reinforcement percentage $\rho_{x}$ was multiplied by the respective yield stress $f_{x}$. This resulted in three new features, denoted by $\rho f_{x}$, where x=“yjt”, “yb”, and “yc” for the joint, beam, and column, respectively. Furthermore, the heights of the beam and column where combined into a single feature by obtaining the ratio $h_{b}/h_{c}$. Thus, the original set of 13 independent variables was reduced to only 7. Finally, in 8 out of 486 instances (1.65%) the compressive strength of the concrete was over 100 MPa. These are considered extreme values for this feature [5] and, thus, this small subsample was removed from the dataset.

**Figure 1:**
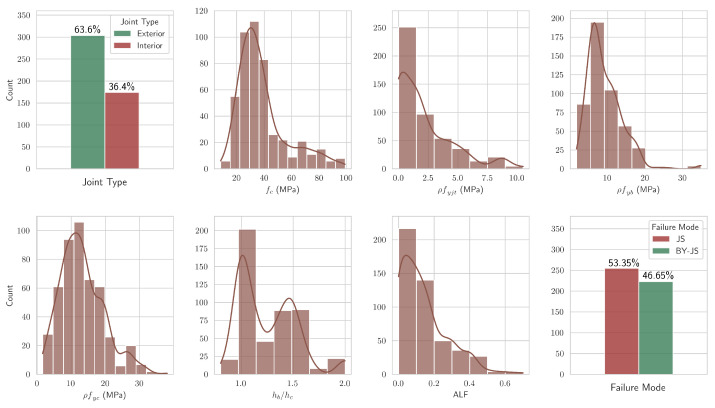
The distribution of the input features and the failure mode in the final dataset, after feature engineering.

**Figure 2:**
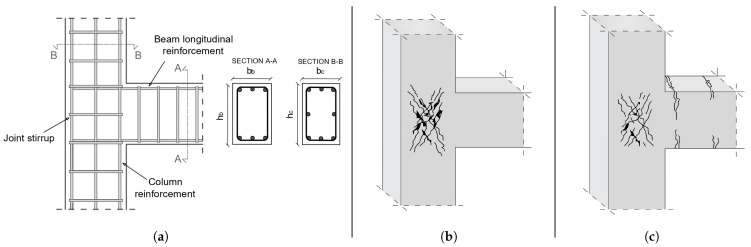
(**a**) Indicative beam–column joint, (**b**) JS failure mode, and (**c**) BY-JS failure mode.

Figure 1 shows the distribution of the attributes that were included in the final dataset after feature engineering. It can be observed that approximately 53.35% of specimens belonged to the failure mode “JS” and the remaining 46.65% belonged to “BY-JS”. A large gap between the two would indicate the presence of what is known as the class imbalance problem [26,27]. This can lead to the degradation of the performance of any ML model, and specialized techniques exist in the literature to address it [28,29]. However, in the present study, the distribution of the failure modes was balanced and, thus, the implementation of such techniques was not necessitated. Finally, Figure 2 presents an indicative RC beam–column joint, as well as the two considered failure modes, for illustration.

### 2.2. Machine Learning Modeling

There are a large number of readily available classification algorithms that can be implemented for the task of the prediction of the failure mode of joints [30,31]. However, as was previously mentioned, the goal of the present study was not to exhaustively search the many available algorithms to identify the best possible machine learning model. Rather, the aim was to utilize the predictions of one such model, in conjunction with the SHAP values explainability technique, as a basis to produce simplified, analytical equations for the prediction of the failure mode. As is shown in the following, the SHAP values approximate the behavior of the underlying ML model. Given that they were fundamental to the proposed formulation, their reliability had to be ensured and thus, it was important for the underlying ML model to obtain a sufficiently high accuracy. To this end, there exist many readily available classification algorithms in the literature.

In the present study, the so-called eXtreme Gradient Boosting (XGBoost) [32] algorithm was employed, which has been successfully utilized in similar studies [2,21]. The fundamental idea of this algorithm is to combine a group of so-called “weak learners” (usually decision trees) in a powerful ensemble model via an iterative procedure called boosting [33]. Thus, the final model takes the form of the following Equation (1) [32,33]:

(1) $$f\left(x\right)=\sum_{m=1}^{N_{E}}\lambda_{m}f_{m}\left(x;\theta_{m}\right)$$

In the above equation, $f_{m}$ are the base models of the ensemble, $\theta_{m}$ and $\lambda_{m}$ are the learned model parameters as well as the respective weights, and $N_{E}$ is the maximum number of base models in the ensemble and is one of the user-defined hyperparameters. In the present study, a maximum number of $N_{E}=10$ trees were employed, each with a maximum depth of 3 and a maximum number of leaves equal to 5. These were selected using a trial-and-error approach, in order to obtain a sufficiently high accuracy without overfitting.

In addition to XGBoost being a very powerful algorithm, it offers several advantages in comparison to other popular classification algorithms, such as artificial neural networks (ANNs) or support vector machines (SVMs). Firstly, as a tree-based algorithm, XGBoost is not susceptible to features whose ranges have different orders of magnitude, which is why no feature scaling was performed as part of the preprocessing presented in the previous section. Secondly, the tree-based nature of XGBoost offers significant computational gains. On the one hand, the ML models themselves are faster to train. On the other hand, the computation of the SHAP values, which generally can be a slow process, can be efficiently carried out using the dedicated TreeSHAP algorithm [34].

### 2.3. Shapley Additive Explanations (SHAP Values)

SHapley Additive exPlanations (SHAP values) [35] belong to the class of the so-called machine learning interpretability methodologies [36], which aim to explain and analyze how complex ML models arrive at their predictions. In addition, SHAP values belong to the subclass of the so-called additive feature attribution methods [35]. Fundamentally, this means that the underlying trained ML model *f* is locally (at each input vector x) approximated by a linear function *g*, which is given by the following Equation (2) [35]:

(2) $$g\left(x^{\prime }\right)=\phi_{0}+\sum_{i=1}^{k}\phi_{i}x_{i}^{\prime }$$

In the above equation, *k* is the number of input variables, $\phi_{i}$ are the corresponding SHAP values, $\phi_{0}$ is the average prediction of the model, and $x^{\prime }\in {\left\{0,1\right\}}^{k}$ is the so-called “simplified input” [35] and shows whether the value of each particular feature is missing or not in the input vector x. The above condition is known as local accuracy [35] and ensures that the sum of the SHAP values is close to the predictions of the underlying ML model. Thus, if this model has a sufficiently high accuracy, the sum of the SHAP values will, in turn, approximate the real target variable in the dataset.

Lundberg and Su-In, who introduced SHAP values in their seminal paper [35], based the computation of these coefficients, $\phi_{i}$, on so-called Shapley regression values. These coefficients were introduced by Lloyd Shapley in the framework of cooperative game theory [37]. Intuitively, the aim is to divide the “score” from a “game” fairly amongst the “players”, according to each “player’s” contribution. Formally, and following the original notation in [35], let F={1,2,…,k} denote the set of all the independent variables/features in the dataset and let S⊆F be a subset, called a “coalition” [38]. Then, the coefficients $\phi_{i}$ are given by the following Equation (3) [35]:

(3) $$\phi_{i}=\sum_{S\subseteq F\setminus \{i\}}\frac{|S|!\left(|F|-|S|-1\right)!}{|F|!}\left(f_{S\cup \{i\}}\left(x_{S\cup \{i\}}\right)-f_{S}\left(x_{s}\right)\right).$$

Thus, conceptually, SHAP values are obtained as a result of a weighted averaging over all feature coalitions of the difference between the model predictions with and without the inclusion of the feature *i*.

In a binary classification setting utilizing XGBoost, as is the case in the present study, SHAP values decompose the so-called log-odds, or logits [34]. Specifically, one of the failure modes is designated as the generic “positive” class, while the other becomes the “negative” class. The log-odds are then given by the following equation [39]:

(4) $$\ln\left(\frac{p}{1-p}\right)$$

In the above equation, *p* denotes the probability that a given input vector belongs to the “positive” class. In the present study, the failure mode “JS” was designated as the positive class and “BY-JS” as the “negative” one.

## 3. Results

In this section, the main results and findings of the present study are presented. As was previously mentioned, the ultimate goal of the proposed formulation was to derive simplified, analytical expressions for the prediction of the failure mode. This was carried out by unraveling the predictions of a fully trained ML classifier via the SHAP values explainability methodology, which was presented in the previous section. Subsequently, these SHAP values were used as a basis for the derivation of the final analytical equations.

As was mentioned, the underlying ML classifier needed to exhibit a high enough accuracy, in order to ensure the reliability of the SHAP values and, ultimately, of the analytical equations. To this end, the rest of this section is organized as follows. Firstly, Section 3.1 briefly presents the results of the XGBoost classifier presented in Section 2.2. Similar results have been analyzed in the literature [2,21]. However, they serve as the basis for the subsequent derivation of the analytical equations, which is the main novelty of the present study. This procedure is presented in Section 3.2, along with the corresponding classification metrics from the derived equations.

### 3.1. XGBoost Classification Results

To measure the performance of the binary classification model, several well-known metrics were employed. Specifically, we utilized the so-called precision, recall, F1-score, and overall accuracy. In a binary classification framework, these are given by the following equations [40]:

(5) $$\begin{array}{c} \mathrm{Precision}=\frac{\mathrm{TP}}{\mathrm{TP}+\mathrm{FP}} \end{array}$$

(6) $$\begin{array}{c} \;\mathrm{Recall}=\frac{\mathrm{TP}}{\mathrm{TP}+\mathrm{FN}} \end{array}$$

(7) $$\begin{array}{c} \;F1\text{-}\mathrm{Score}=2\frac{\mathrm{Precision}\cdot \mathrm{Recall}}{\mathrm{Precision}+\mathrm{Recall}} \end{array}$$

(8) $$\begin{array}{c} \;\mathrm{Accuracy}=\frac{\mathrm{TP}+\mathrm{TN}}{\mathrm{TP}+\mathrm{TN}+\mathrm{FP}+\mathrm{FN}} \end{array}$$

In the above equations, TP (true positive) denotes the number of input vectors that belong to the generic “positive” class (in this case, the failure mode “JS”) and are correctly assigned to it by the classifier, TN (true negative) denotes the number of input vectors that belong to the generic “negative” class (in this case, the failure mode “BY-JS”) and are correctly classified, while FP (false positive) and FN (false negative) denote the input vectors that the model incorrectly assigns to the respective failure modes. It should be highlighted that the first three metrics are defined for each failure mode separately, while accuracy is an overall metric.

In order to obtain the performance metrics, a so-called *k*-fold cross-validation scheme was employed [41]. This procedure started by splitting the dataset into *k* parts. Subsequently, the algorithm iterated over these partitions and, at each iteration, it utilized the k−1 parts of the dataset for training, while the remaining part was used to gauge the performance of the trained model on “unseen” data. The final classification metrics were obtained as the averages of the respective metrics of each fold.

This procedure greatly reduced the variance of the obtained classification metrics, which can occur due the randomness of the train/test split. Thus, the reliability and robustness of the obtained results was increased. In the present study, k=10 folds were employed. Furthermore, it should be highlighted that the cross-validation scheme was stratified [41]. This means that each train/test subset had approximately the same proportion of exterior/interior nodes and failure modes and these proportions were approximately the same as those in the original dataset.

**Figure 3:**
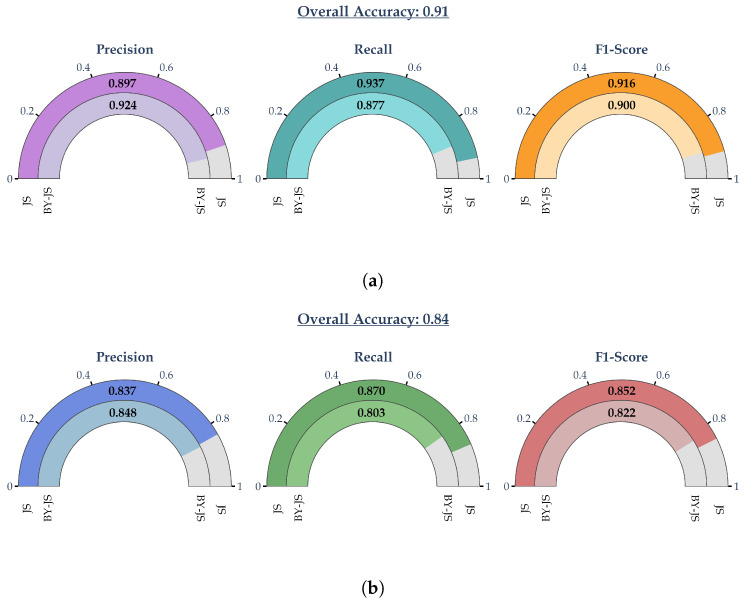
Classification metrics of XGBoost classifier obtained via 10-fold cross-validation. (**a**) Train dataset and (**b**) Test dataset.

Following the above, Figure 3 presents the cross-validated classification metrics obtained from the XGBoost classifier described in Section 2.2. As can be readily observed, the average classification accuracy using the test dataset was high, approximately 84%. The respective classification metrics of the two failure modes were relatively close to each other, indicating a model that was not skewed towards one of the two modes. This was also supported by the fact that the precision, recall, and F1-score were all relatively close to each other and to the overall accuracy.

### 3.2. Derivation of Analytical Equations

In this section, the main results and findings of the present study are presented. These pertain to the derivation of simplified, analytical equations for the rapid and efficient determination of the failure mode of reinforced concrete beam–column joints. These equations are based on the SHAP values presented in Section 2.3, and they can be constructed by sequentially and incrementally adding more terms to achieve the desired tradeoff between complexity and accuracy [22].

To this end, the first step in the proposed methodology pertained to the computation of the SHAP values, $\phi_{i}$, for each input vector. These were computed separately for the train and test parts of each fold in the 10-fold cross-validation scheme. This facilitated the training of the analytical equations in a similar manner to the underlying ML model. Subsequently, the SHAP values were aggregated and normalized to produce a single quantifier for the overall effect of each feature on the model’s predictions. Specifically, these aggregated values were obtained as follows [42]:

(9) $${\bar{\phi }}_{j}=\frac{\phi_{j}^{\prime }}{\sum_{m=1}^{k}\phi_{k}^{\prime }},\;\phi_{j}^{\prime }=\frac{1}{n}\sum_{i=1}^{n}\left|\phi_{ij}\right|$$

**Figure 4:**
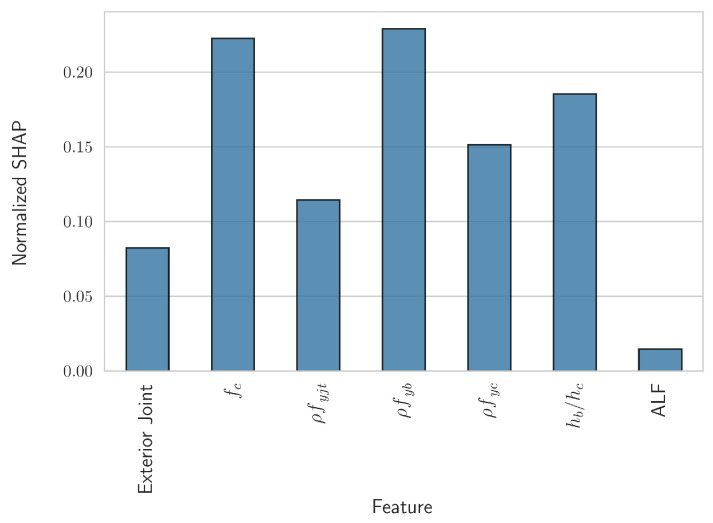
Cross-validated normalized SHAP values, ${\bar{\phi }}_{j}$, as in Equation (9).

In the above equation, $|\phi_{ij}|$ denotes the SHAP value corresponding to the feature *j* computed for the input vector *i*, *n* is the total number of such input vectors in the dataset, and *k* is the total number of independent variables/features. Due to the above normalization, the extracted coefficients could be interpreted as the percentage of the average influence of each feature on the model’s predictions. The coefficients ${\bar{\phi }}_{j}$ were computed for each fold in the 10-fold cross-validation and their average was obtained. The results are shown in Figure 4.

It can be readily observed that the two most important numerical features were found to be $f_{c}$, with an average importance of approximately 22.3%, and ${\rho f}_{yb}$, with an average importance of approximately 22.9%. The ratio of the height of the beam to the height of the column also had a relatively high importance, while the importance of the axial load factor was found to be relatively small.

As was previously discussed, the fundamental idea of the proposed methodology was to fit simplified, analytical equations to the SHAP values of each feature. These values were additive and, according to the local accuracy condition shown in Equation (2), their sum approximated the true predictions of the ML model. Thus, as was discussed in Section 2.3 and Equations (3) and (4), they approximated the logit of the probability that a given input vector belonged to the failure mode “JS”.

**Figure 5:**
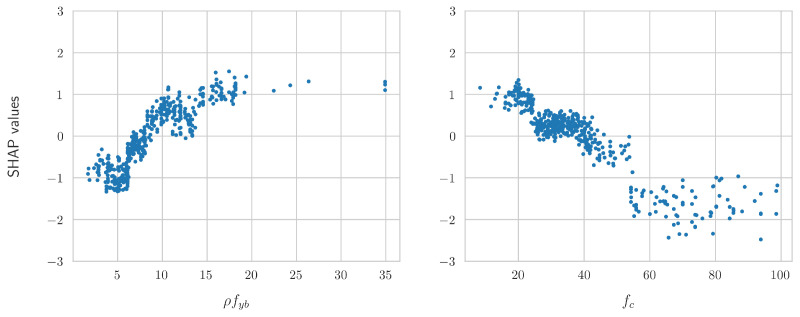
Scatter plots of the SHAP values of the two most significant numerical features, namely ${\rho f}_{b}$ and $f_{c}$.

Figure 5 presents the scatter plots of the SHAP values for the two most important features as identified in Figure 4, namely ${\rho f}_{yb}$ and $f_{c}$. The analytical equations that corresponded to the contribution of each feature were derived based on these scatter plots. For example, the SHAP values of ${\rho f}_{yb}$ could be approximated by a square root function, a logarithm, or a parabola. Similarly, the SHAP values of $f_{c}$ could be approximated by a function of the form 1/x or an exponential.

**Figure 6:**
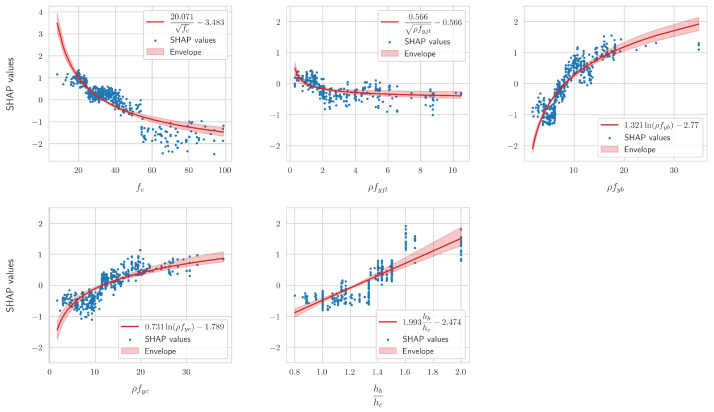
Scatter plots of the SHAP values of the numerical features that were employed, along with the best-fitted cross-validated curves. The envelope corresponds to the minimum and maximum values across the 10 folds.

In the present study, we experimented with a wide array of possible analytical functions for each feature, including polynomials of various degrees, and the aforementioned roots, logarithms, and inverses. For each feature, the form of the analytical equation that resulted in the highest accuracy was selected. The final coefficients in each case were computed by averaging the coefficients that were computed for each fold in the 10-fold cross-validation process. Figure 6 shows the scatter plots of the SHAP values of the features that were employed in the final equations, as well as the best-fitted curves that resulted from the cross-validation process. It also displays the envelope of each fitted curve, i.e., the minimums and maximums across the 10 folds. It can be readily observed that the coefficients of the fitted curves did not exhibit a huge variation across each fold, as the respective bands of the envelopes are not very large. In any case, as was previously mentioned, the 10-fold cross-validation procedure reduced the variance of the coefficients that were obtained for each particular dataset.

The curves presented above are in qualitative agreement with the currently established literature with regards to the behavior of reinforced concrete beam–column joints. For example, increasing the compressive strength of the concrete and the amount or strength of the stirrups in the joint decreases the log-odds that the given joint/input vector will exhibit the “JS” failure mode, i.e., that it will belong to the “positive” class. Additionally, increasing the amount of reinforcement in the beam or its strength results in a beam with increased strength compared to the joint, thus increasing the likelihood that the “JS” failure mode will occur. A similar observation holds for increasing the ratio of the height of the beam, compared to the height of the column. It should be noted that there is a correlation between the height of each bar in Figure 4 and the range of the respective SHAP values in the above figure. Furthermore, the variations in these values, as seen, for example, in the subplot for $f_{c}$, can be further analyzed using SHAP interaction values [34]. However, due to the excellent results achieved herein, this added complexity was not deemed justifiable. Before the final forms of the derived analytical expressions are presented, two additional points should be highlighted.

In addition, as can be observed from Figure 5, the expression for the contribution of ${\rho f}_{yjt}$ was not valid if ${\rho f}_{yjt}=0$, i.e., if the joint had no stirrups. For those cases, the SHAP values of this feature ranged from approximately 0.36 to 0.505. Therefore, its contribution could be adequately captured using a single value, the mean. In this case, the average across the 10 folds was approximately equal to 0.429, with a standard deviation of approximately 0.055. Furthermore, as can be seen from Equation (2), in order for the condition of local accuracy to be valid, the constant $\phi_{0}$ had to be added. This corresponded to the mean model prediction. In our case, this had an average value of approximately 0.156 across the 10 folds and a standard deviation of approximately 0.0067. It should be reiterated that these values correspond to the log-odds, as defined in Equation (4), that a given input vector/joint belonged to the failure mode “JS”. Thus, if the inverse sigmoid transformation is employed to solve for the actual probability *p*, we obtain

$$p=\frac{1}{1+e^{-0.156}}\approx 0.5389.$$

This means that, on average, the model predicted a 53.89% probability for the “JS” failure mode, which was close to the distribution of the original dataset, as shown in the last subplot of Figure 1. Following the above, the final derived analytical equations for the prediction of the failure mode took the following forms. Specifically, for joints with stirrups, i.e., wherein ${\rho f}_{yjt}>0$, we have

(10) $$\frac{20.071}{\sqrt{f_{c}}}+1.321\ln{\rho f}_{yb}+0.731\ln{\rho f}_{yc}+1.993\frac{h_{b}}{h_{c}}+\frac{0.566}{\sqrt{{\rho f}_{yjt}}}-10.926,$$

while for joints without stirrups, i.e., wherein ${\rho f}_{yjt}=0$, we have

(11) $$\frac{20.071}{\sqrt{f_{c}}}+1.321\ln{\rho f}_{yb}+0.731\ln{\rho f}_{yc}+1.993\frac{h_{b}}{h_{c}}-9.931$$

The above equations were utilized to predict the failure mode of a joint as follows. First, the geometric and mechanical characteristics of the beam, column, and joint were obtained. These included their dimensions as well as the amount and strength of the reinforcement bars. Subsequently, Equations (10) and (11) were utilized. If the resulting quantity was ≥0, then the corresponding probability was ≥0.5. Thus, the joint under consideration was predicted to belong in the “JS” failure mode. Otherwise, it was assigned to “BY-JS”.

**Figure 7:**
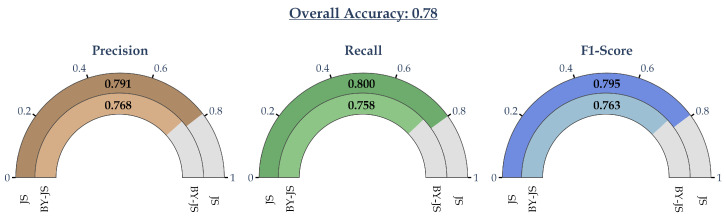
The classification metrics of the entire dataset of the final fitted Equations (10) and (11) using the cross-validated coefficients.

Table 1 shows the respective average classification metrics obtained via the 10-fold cross-validation procedure. For each fold, the classification metrics derived from the corresponding analytical equation were computed separately using the respective training and test subsets. The respective standard deviations are also presented. Finally, Figure 7 displays the classification metrics of the final model, i.e., wherein the formulas in Equations (10) and (11) were employed.

As can be readily observed from Table 1, the average classification metrics between the training and test datasets were close to each other. This indicates that the fitted analytical equations did not overfit. This can be attributed to the fact that relatively simple terms were selected for the analytical equation of each feature, as shown in Figure 6. In addition, as was mentioned in Section 2.2, the employed cross-validation scheme was stratified, which means that each train/test subset had approximately the same proportion of exterior/interior nodes and failure modes, and these proportions were approximately the same as those in the original dataset. This meant that the training and test subsets were balanced, which could also contribute to reducing overfitting.

Similarly, from Figure 7, it can be readily observed that the final simplified equations achieved high classification metrics in both failure modes. For the failure mode “JS”, the precision, recall, and F1-score were all very close to each other and approximately equal to 80%. This demonstrated the very good ability of the analytical equations to detect this failure mode, which, as was mentioned, was the most severe and potentially damaging one. Similarly, the precision, recall, and F1-score for the “BY-JS” failure mode were also very close to each other, ranging from approximately 76–77%. These metrics were approximately 2.3–4.4% lower than the corresponding ones of the “JS” failure mode. If these differences were large, then the derived model would be considered biased towards the failure mode with the highest metrics. However, in this case, the respective differences were very small and the derived model was balanced between the two modes. Finally, it should be highlighted that the obtained overall accuracy was also high, approximately equal to 78%. All the aforementioned metrics, including the overall accuracy, were relatively close to the respective metrics obtained by the underlying XGBoost model. Thus, the derived Formulas (10) and (11) could be employed confidently, without a substantial performance degradation, compared to the underlying fully trained ML model.

The above results pertained to the “balanced” classifier obtained in Equations (10) and (11). The classification threshold for the “JS” failure mode was set at p≥0.5 or, equivalently, $\ln\left(\frac{p}{1-p}\right)\geq 0$. However, it is often the case, especially in engineering applications, that “erring on the side of safety” is preferred. For example, it could be required that a higher recall is obtained for the “JS” failure mode, the most dangerous and potentially damaging one. This would lead to more “JS” specimens being identified, although the corresponding precision index would be lower. This is due to the well-known precision–recall tradeoff [43,44]. This tradeoff can be visualized via so-called precision–recall (P-R) curves [45,46]. Similar to ROC curves [47,48], the area under the curve (AUC) of P-R curves gives a quantification of their overall performance. The baseline in this case is the simple “majority classifier”, i.e., a model that constantly assigns the input vectors to the class with the most input vectors in the training dataset.

**Figure 8:**
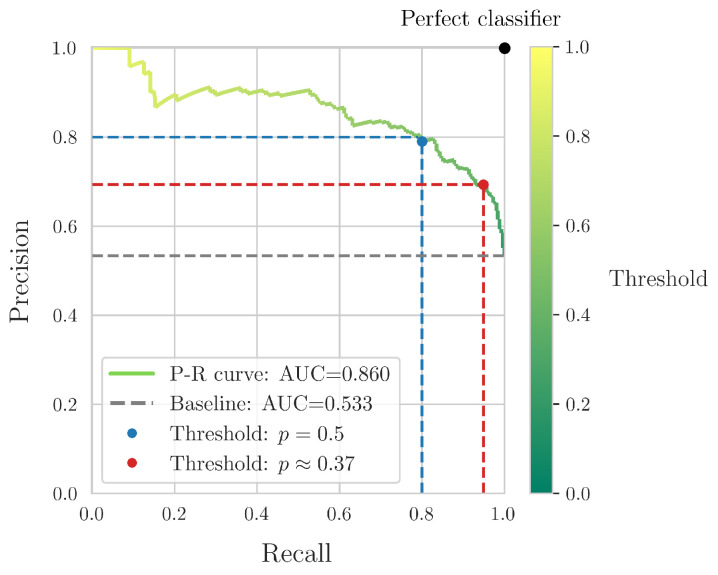
Precision-Recall curve for the classifier obtained via the analytical Equations (10) and (11).

Following the above, Figure 8 shows the precision–recall curve of the “JS” failure mode, computed via the analytical Equations (10) and (11). The top-right corner corresponds to the “perfect classifier”, and the closer the P-R curve is to it, the better its overall performance was.

Firstly, it can be readily observed that as the threshold for *p* decreased, more input vectors were assigned to the “JS” failure mode. Thus, the recall index of this mode increased, while, conversely, the precision index decreased. The threshold p=0.5 was a balanced one, with the precision and recall very close to each other and close to 80%. We also identified the *p* threshold for a recall of ≈95%, which is a commonly employed engineering standard. In this case, p≈0.37 and the precision was reduced to ≈69%, while the overall accuracy of the classifier was reduced from ≈78% to ≈75%.

## 4. Summary and Conclusions

The mechanical behavior of reinforced concrete beam–column joints during a major seismic event is one of the most critical aspects that affect the overall behavior of a structure. Qualitatively, modern design codes dictate that, if failure is to occur, the flexural yielding of the beam, which is a ductile failure mode, should be the first to occur (“BY-JS” failure mode) and not the brittle shear failure of the joint (“JS” failure mode). Thus, the rapid and reliable estimation of the joint failure mode is one of the most crucial engineering problems faced by modern societies.

In this regard, machine learning classification algorithms have a demonstrated capability to produce state-of-the-art results in similar engineering challenges. However, they often suffer from a lack of transparency, as their predictions cannot be easily understood. Furthermore, ML algorithms are hard to incorporate into adopted engineering practice and design codes, which necessitate the existence of simplified analytical formulas in order to make predictions.

Thus, in the framework of the present research effort, a methodology for the derivation of simplified analytical equations for the prediction of the failure mode of reinforced concrete beam–column joints is presented. The proposed formulation aimed to create a link between the proven ability of ML algorithms to produce state-of-the-art results and the aforementioned need for simplified analytical expressions.

To this end, an underlying ML model was employed for the classification task, i.e., for the prediction of the probability corresponding to each failure mode. Subsequently, the SHAP values explainability technique was utilized to decompose these predictions into components pertaining to each individual feature. As Equation (9) and Figure 4 demonstrate, this allowed for the quantification of the overall influence of each feature on the outcome. Guided by this feature-importance hierarchy, the most important features’ SHAP values were utilized as a basis for the derivation of the simplified analytical expressions. The proposed methodology offered several distinct advantages.

Firstly, the derived equations were derived in an informed manner, wherein more terms could be added or removed independently to achieve the desired tradeoff between complexity and accuracy, guided by the feature-importance hierarchy presented in Figure 4. This was also facilitated by individually adjusting the form of the analytical equation that was fitted to each feature’s SHAP values, guided by the form of the corresponding SHAP scatter plots, as shown in Figure 5.

Secondly, as is shown in Equation (2), the SHAP values approximated the behavior of the underlying ML model. Thus, the derived equations could achieve comparable levels of performance, as demonstrated in Figure 3b and Figure 7. Indeed, the XGBoost classification model achieved an overall accuracy of approximately 84%. It corresponding precision, recall, and F1-score for the “JS” failure mode ranged from approximately 83.7 to 87%. Accordingly, the overall classification accuracy for the derived Equations (10) and (11) was approximately 78% and the corresponding classification metrics ranged from 79.1 to 80%.

Finally, it should be noted that the obtained classification metrics of the analytical equations were balanced between the two failure modes. Indeed, as is shown in Figure 7, the respective differences ranged from approximately 2.3 to 4.4%. However, as is demonstrated by Figure 8, the derived equations could easily be modified to achieve a different metric balance, for example, to obtain a higher recall index for the “JS” failure mode.

Overall, the results presented herein are promising, as they demonstrate the potential for the derivation of analytical expressions with a comparable accuracy to fully trained ML models. Even though the examined application pertained to the prediction of the RC joint failure mode, the results presented here encourage the application of the examined methodology to similar complex and impactful engineering challenges. Finally, even though the derived equations are based on a dataset consisting of 478 RC joint experimental results, they can be continuously updated as more data are incorporated, potentially leading to new insights into the behavior of RC beam–column joints.

## Figures and Tables

**Table 1:** Cross-validated classification metrics of fitted analytical curves of each fold.

	Training Set		Testing Set	
	BY-JS	JS	BY-JS	JS
**Precision**	0.7697±0.016	0.7932±0.010	0.7749±0.071	0.7847±0.056
**Recall**	0.7613±0.014	0.8004±0.018	0.7447±0.080	0.8072±0.071
**F1-Score**	0.7653±0.011	0.7967±0.012	0.7575±0.065	0.7944±0.054
**Accuracy**	0.7822±0.011		0.7781±0.057	

## Data Availability

The data that support the findings of this study are available upon reasonable request.

## Abbreviations

The following abbreviations are used in this manuscript:

RC	Reinforced concrete
ML	Machine learning
ANN	Artifical neural network
SHAP	SHapley Additive exPlanations
*k*-NN	*k*-nearest neighbors
SVM	Support vector machine
OLS	Ordinary least squares
XGBoost	eXtreme Gradient Boosting
P-R	Precision–recall
AUC	Area under the curve
